# Impact of nocturnal hemodialysis on peripheral uremic neuropathy

**DOI:** 10.1186/s12882-015-0133-2

**Published:** 2015-08-12

**Authors:** Sassan Ghazan-Shahi, Timothy Jee Kam Koh, Christopher T. Chan

**Affiliations:** Department of Internal Medicine, Division of Nephrology, University Health Network Toronto General Hospital, 200 Elizabeth Street, Toronto, Ontario M5G 2C4 Canada

**Keywords:** Home hemodialysis, Uremic peripheral neuropathy, Intensive hemodialysis

## Abstract

**Background:**

Uremic neuropathy is a common complication in patients with end stage kidney disease. Its pathogenesis has been attributed to accumulation of uremic toxins. Kidney transplantation has been the best therapeutic option.

**Case presentation:**

We describe a case of severe uremic peripheral neuropathy, which improved after conversion from a conventional renal replacement therapy to nocturnal hemodialysis.

**Conclusion:**

Enhanced uremic control by intensive hemodialysis may contribute at least in part to clinical and neurophysiological improvements of uremic neuropathy.

## Background

Uremic peripheral neuropathy is a common condition amongst patients with end stage kidney disease (ESKD), which in most cases is progressive, and in some significantly debilitating. At present, renal transplantation remains the best therapeutic option for uremic neuropathy [1, 2].

We report the clinical and electrophysiologic findings in a man with this disorder who demonstrated significant improvement after conversion to nocturnal hemodialysis.

## Case Report

A 48 year old man with ESKD presented with severe decreased sensation in his bilateral feet, originated from bottom of his feet, and gradually advanced to above the ankle level over the course of 2–3 years.

He reported the sensation of “walking on cushions”, as well as progressive difficulty with ambulation. Additionally, he complained of ongoing pain in bilateral legs, as well as dysaesthesia in bilateral hands. He had been diagnosed with ESKD at age 37, secondary to poorly controlled hypertension. He was initiated on peritoneal dialysis (PD) therapy for two years, before receiving a living-related-donor kidney transplant at age 39. His renal graft failed due to chronic allograft nephropathy after 7 years, when he had to be restarted on PD for renal replacement therapy. He had been on PD therapy for 4 years at the time of presenting with above symptoms. He had minimal residual urine output during the last 2 years of PD. As a result, he was receiving continuous cycling peritoneal dialysis (CCPD), 8 liters over 9 hours overnight, with a last fill of 1.5 L, with good compliance. Membrane transport type with 4-hour peritoneal equilibrium test (PET) was high average. His past medical history included a remote history of malarial infection, gout and native kidney nephrectomy due to findings of a mass compatible with renal cell carcinoma on post-operative pathology report, one year prior to current presentation.

He also had been diagnosed with type 2 diabetes mellitus one year prior to the above presentation, and had appropriate glycemic control achieved by diet and lifestyle modifications. He denied any history of thyroid disease, vitamin deficiencies, lyme disease, alcohol abuse, chemotherapy, heavy metal exposure, amyloidosis or other systemic `diseases. There was no previous history of peripheral vascular disease. His medications included prednisone, verapamil, calcium carbonate, atorvastatin, darbepoetin injection, multivitamin tablet and venlafaxine. Cyclosporine and Mycophenolate Mofetil had been discontinued for 3 years prior to the above presentation.

On physical examination, his vital signs were stable; examination of head and neck, heart, lungs and abdomen was unremarkable. On neurologic exam, there was no evidence of muscle atrophy or fasciculations. The deep tendon reflexes were absent throughout. Sensory exam was significantly abnormal; pinprick and light touch sensations were impaired up to level of mid-calf bilaterally, there was moderate deficit of joint position sensation at the toes. Vibration thresholds were measured 50 microns on right great toe, and 38 microns on the left. Gait was prominently antalgic, but otherwise without specific characteristics. Muscle strengths were found to be symmetrical and 5/5 bilaterally. Cranial nerves were intact. The rest of physical exam was unremarkable.

His initial laboratory testing showed abnormalities consistent with ESKD: blood urea nitrogen above 10 mmol/L and serum creatinine above 1000 umol/L, for the previous several years. HbA1C had been less than 7 % consistently. Hemoglobin averaged around 100 g/L, potassium 3.7 mmol/L, corrected calcium 2.4 mmol/L and phosphate 1.7 mmol/L. Average weekly Kt/V for PD, as an adequacy index was 2.84.

Nerve conduction studies were performed, results of which were compatible with a severe, length-dependent, combined demyelinating and axonal, sensorimotor polyneuropathy. This was deemed to be most in keeping with uremic neuropathy. Although the patient had been diagnosed with diabetes the year before this presentation, the short duration of the disease and absence of other complications of diabetes, made the diagnosis of diabetic neuropathy unlikely. Given the patient’s severity of clinical symptoms , despite a reasonable accepted adequacy for peritoneal dialysis, it was decided to convert his renal replacement therapy to Nocturnal Home hemodialysis (NHD) 5–6 nights per week, 7–8 hours per session of dialysis. We hypothesize that an increase in uremic clearance may improve his uremic neuropathy.

Within 6 months following the switch to NHD, the patient reported significant improvement of dysaesthesia, with reduction in leg pain and improvement in his ambulation.

Routine laboratory values showed improvement in dialysis parameters, including average hemoglobin of 121 g/L, potassium of 4 mmol/L, creatinine of 540 mmol/L, blood urea nitrogen of 10 mmol/L, corrected calcium of 2.40 and phosphate of 0.8 mmol/L.

Repeat sensory and motor nerve conduction studies were performed 1 and 2 years after conversion to NHD; there were improvements in nerve conduction velocity, amplitude and latency measures, in both sensory and motor components. The neurophysiological improvements were more pronounced in the upper extremities. These findings were consistent with resolution of symptoms.

**Fig. 1:**
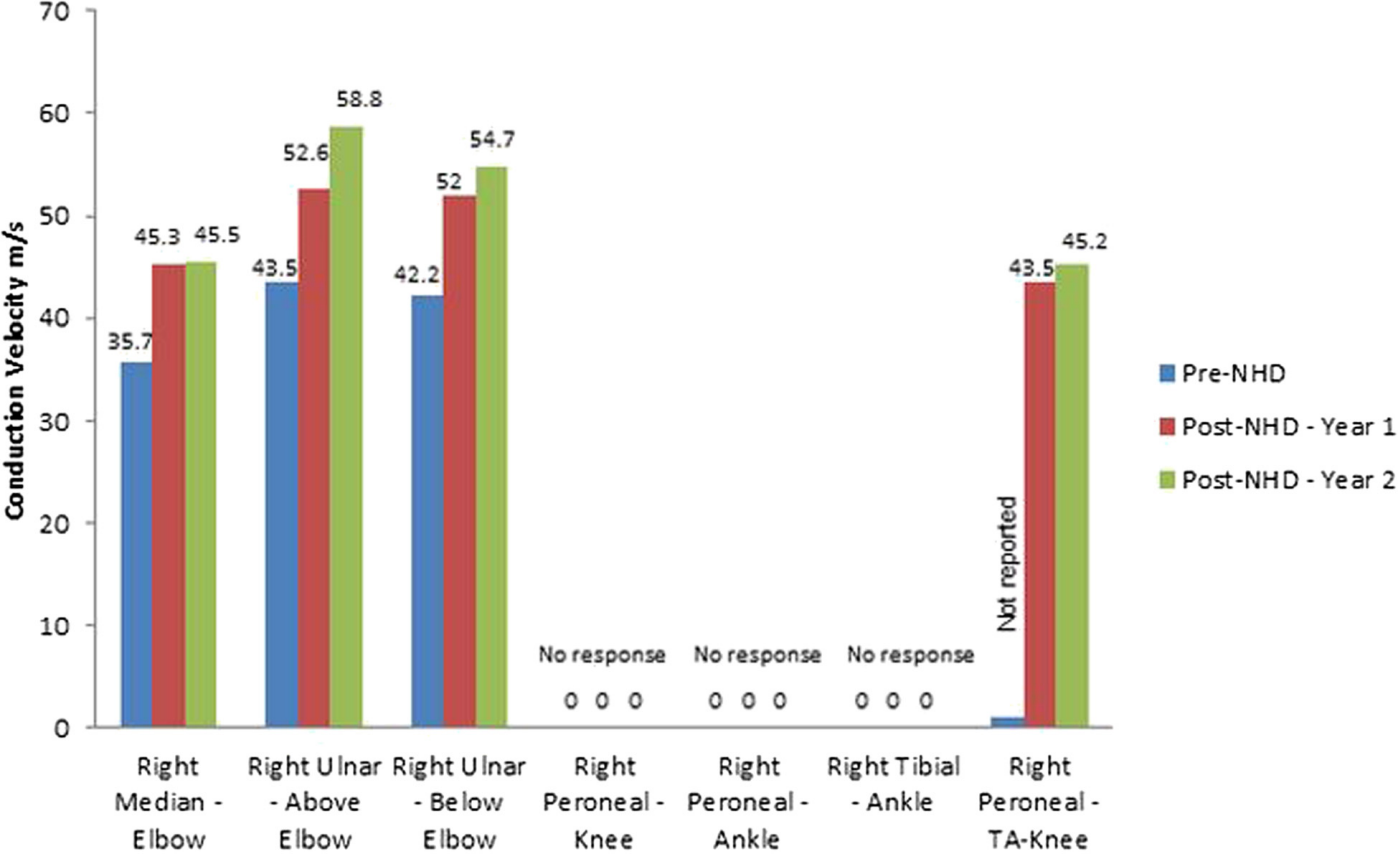
Motor nerve conduction velocities. Before conversion to NHD, 1 year after conversion and 2 years after conversion

**Fig. 2:**
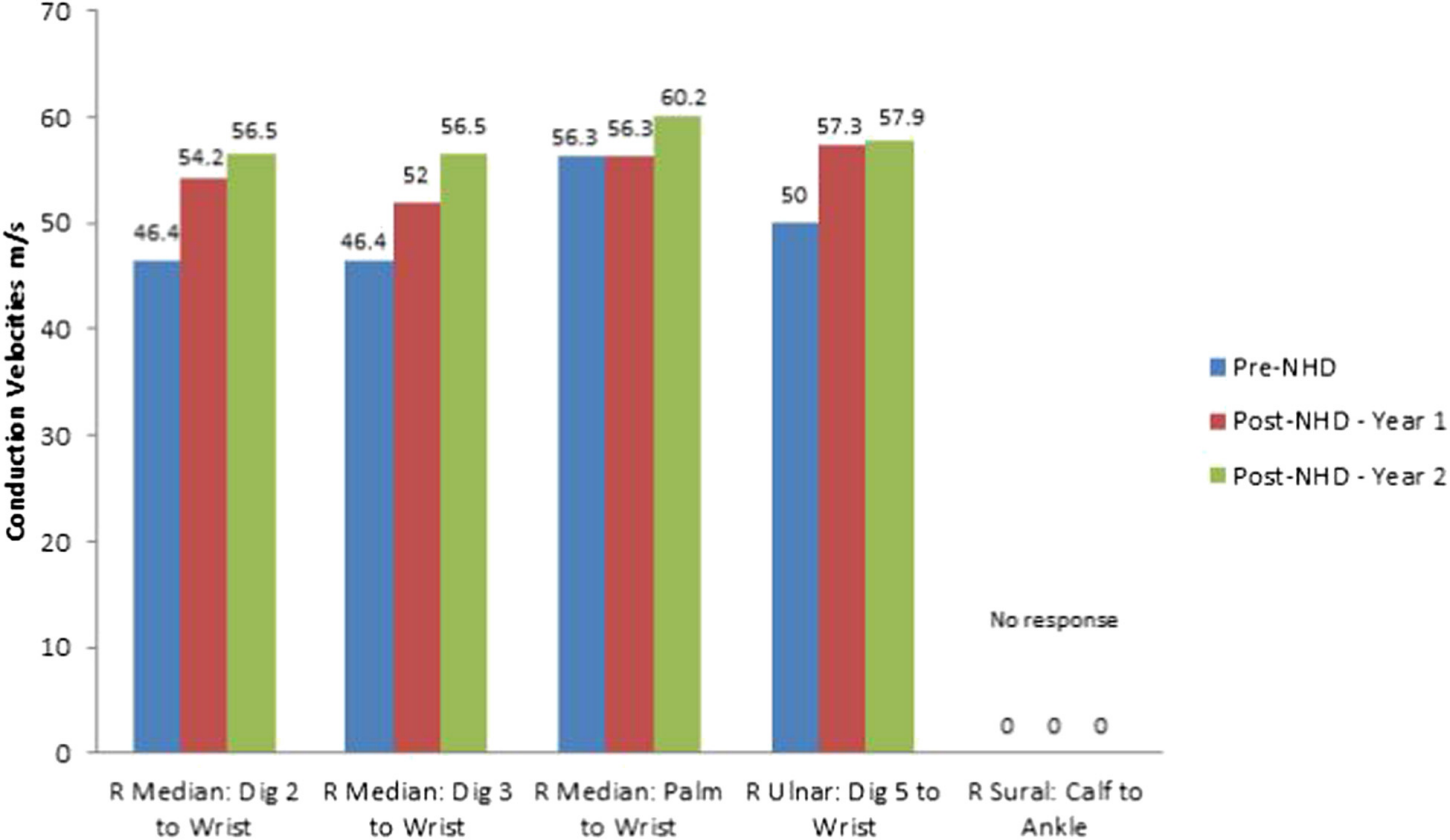
Sensory nerve conduction velocities. Before conversion to NHD, 1 year after conversion and 2 years after conversion. (R: Right)

Figures 1 and 2 demonstrate motor and sensory nerve conduction studies at baseline and after conversion to NHD.

## Discussion

Uremic neuropathy is a common condition in patients with ESKD. The prevalence ranges from 60–93 % in the dialysis population [3]. In non-dialysis patients with chronic kidney disease (CKD), the prevalence was reported to be 64 % in one study [4].

Uremic neuropathy typically presents as a slowly progressive sensorimotor axonal neuropathy. Other forms of neuropathy described include a rapidly progressive motor neuropathy, and a small fibre neuropathy, which are rarer in presentation. The clinical manifestations of uremic neuropathy may vary. Some patients may notice primarily sensory symptoms early in the disease, which may present as paraesthesia, pain, or loss of sensitivity. Loss of deep tendon reflexes has also been described. With progression, there is increasing motor involvement, with muscle atrophy and loss of strength. Symptoms progress proximally, with involvement of the upper limbs occurring after lower limb involvement. The underlying pathology is believed to be due to a demyelinating process, and is contributed by axonal degeneration and loss.

Various neurophysiological findings are associated with uremic neuropathy, and nerve conduction studies remain the gold standard for its diagnosis. In general, neuropathies that result in axonal degeneration result in conduction velocities that are mildly reduced. Values typically are reduced but remained above the threshold of 30 ms^−1^ in the lower limbs, and 35 ms^−1^ in the upper limbs. In contrast, for demyelinating lesions, values are generally expected to be substantially lower. In addition, the amplitude of evoked potentials is reduced in axonal degeneration, whereas it would be expected to be relatively preserved in demyelinating lesions. Early studies in motor nerve conduction parameters demonstrated a slowing of conduction velocities. Other studies demonstrated a generalized slowing in both sensory and motor nerves, with reductions in sensory response amplitudes [5].

More recently, Krishnan et al. have described a change in sural sensory potential as being the most sensitive nerve conduction abnormality in patients with CKD [6].

Improvements of uremic neuropathy with dialysis and transplantation have been described. Early observational studies demonstrated a reduction in the prevalence of neuropathy with either an increase in frequency or dose of dialysis [7]. As a result, investigators hypothesize that uremic toxin(s) contribute, at least in part, to the pathogenesis of uremic neuropathy. Both peritoneal and conventional hemodialysis have demonstrated partial efficacy in reducing the progression of uremic neuropathy. Recently, hemodiafiltration has been associated with a reduction in motor nerve excitability parameters as compared to patients on high flux hemodialysis, both after a single session, and longitudinally [8]. To date, renal transplantation has been the preferred therapeutic option to provide either a “cure” or an improvement in the severity of uremic neuropathy. As early as the 1970s, Bolton et al. reported functional recovery in 3 out of 10 patients post transplantation, even though residual clinical signs and electrophysiological abnormalities remain [1]. Other patients either had moderate recovery, or subclinical improvement reflected by nerve conduction velocities. Similarly, Hupperts et al. reported two patients that had either partial or complete recovery of uremic neuropathy post renal transplantation [9]. Consistent reports were also reported in the pediatric population [10].

To our knowledge, this is the first description of a patient with uremic neuropathy who demonstrated both clinical and neurophysiological improvements after converting from conventional renal replacement therapy to NHD. Our results add to the existing literature, which suggests that accumulated uremic toxins are responsible for the progression of uremic neuropathy. The use of intensive hemodialysis offers another potential therapeutic option to achieve the necessary clearances associated with amelioration of neurological symptoms. Given that NHD provides augmented uremic clearance in comparison to all forms of conventional renal replacement therapy, it is tempting to speculate that our present case report is a direct result of enhanced toxin removal. Equally important, other authors have proposed the role of physiological maintenance of electrolytes such as potassium. Prolonged hyperkalemia has been proposed as a mechanism that may cause disruption of normal ionic gradients and activate damaging Ca^++^-mediated processes, leading to axonal loss in the setting of ESKD. Krishnan et al. suggest that the maintenance of serum K^+^ within normal limits between dialysis sessions, rather than simple avoidance of hyperkalemia, is likely to reduce the incidence and severity of uremic neuropathy [11, 12]. It is reasonable to propose that a longer and more frequent dialysis regimen should result in a closer approximation of normal physiological control of solutes such as potassium.

## Conclusion

In summary, uremic neuropathy contributes to the morbidity of patients on dialysis. We report the use of NHD in a patient with severe debilitating uremic neuropathy. Prospective evaluation of NHD on clinical evolution of uremic neuropathy is warranted.

## Consent

The patient provided full informed consent for gathering the data and publishing the case.

## Abbreviations

ESKD: End Stage Kidney Disease
PD: Peritoneal Dialysis
NHD: Nocturnal Hemodialysis
CKD: Chronic Kidney Disease
